# Lifetime Prediction of Polymers: To Bet, or Not to Bet—Is This the Question?

**DOI:** 10.3390/ma11081383

**Published:** 2018-08-08

**Authors:** Ignazio Blanco

**Affiliations:** 1 Department of Civil Engineering and Architecture, University of Catania, Viale Andrea Doria 6, 95125 Catania, Italy; iblanco@unict.it; 2 UdR-Catania Consorzio INSTM, Viale Andrea Doria 6, 95125 Catania, Italy

**Keywords:** lifetime prediction, thermal stability, polymer degradation, thermogravimetric analysis, induction period

## Abstract

Polymers are a great and very important category of organic compounds that have changed our lifestyle. In the last eighty years, we have used them for the most varied applications, and from the first structural ones we began to investigate their durability, which can be fatal in the successful completion of the application for which the material was designed. Over the last thirty years, the environmental problems related to the disposal of polymers that have completed their lifecycle have begun to arise, and the need to foresee their end of life has become increasingly urgent. In this manuscript, the reliability of the lifetime predictions of polymeric materials is faced with comparing measurements obtained at low temperature with those carried out at high temperatures, in the molten state. The obtained data were treated by a well-established kinetics model and discrepancies were observed in the two different conditions (high and low temperatures), which led to a mismatching between expected and real data. A correction of the data extrapolated from measurements obtained at high temperatures, by using a novel equation which takes into account the induction period (IP) of the degradation process, is proposed. Considerations about the useful parameters, namely initial decomposition temperature (*T*_i_), activation energy of degradation (*E*_a_), and glass-transition temperature (*T*_g_), to be used for making predictions, are also carried out.

## 1. Introduction

**Figure 1:**
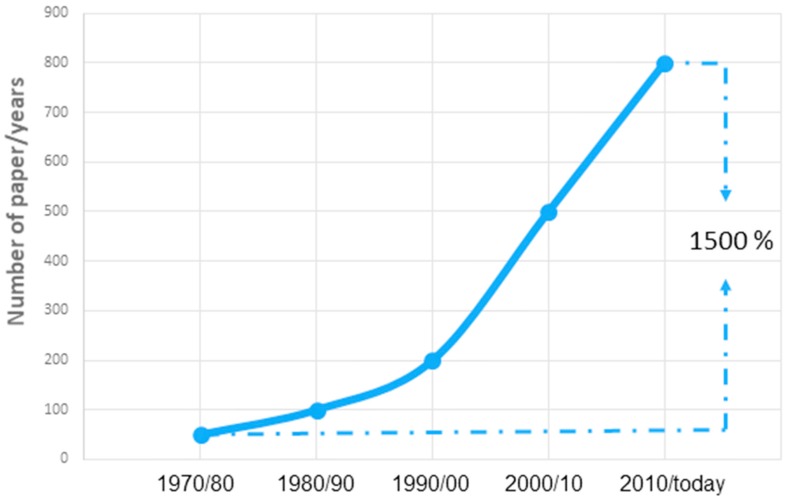
Time evolution of manuscripts published on International Journals having "lifetime prediction" as topic. Source: Scopus (https://www.scopus.com/).

To realize the great interest on this topic, just put the keyword “lifetime prediction” in the Scopus search engine. Since data are available, we can observe an exponential growth of scientific works concerning the lifetime prediction (Figure 1). It is changing from an annual average of fifty manuscripts in the seventy-eighty, at the present day, to an annual average of eight hundred manuscripts, with an increase of 1500 percent. Although it is right to remark that in the same period there has been an overall increase in scientific publications, if we repeat the same operation by entering two other very common search terms, such as “glass transition” or “calorimetry”, a remarkable growth can obviously be observed, but that does not reach even half from publications relating to “lifetime prediction”.

Some years ago, the International Confederation for Thermal Analysis and Calorimetry (ICTAC), realizing this interest, has instituted a special scientific committee in order to set the use of Thermal Analysis (TA) methods for making lifetime predictions by working closely with Kinetics Committee. Furthermore, in 2016, for the first time, during the ICTAC Conference, which was held in Orlando, Florida, a special session on lifetime prediction has been inserted in the congress program that has had wide and geographically variegated participation [1].

Many aspects of the lifetime prediction were treated and some interesting conclusions were drawn. First of all, sixty percent of the presentations covered polymeric materials, highlighting that thermo-oxidative aging of polymers is one of the most serious problems felt by the scientific community. One of the addressed issues was the role of aging and the deteriorating of physical and mechanical properties, for instance of rubber during usage. It is a very serious problem. Do you remember Challenger? The space shuttle exploded shortly after launch on 28 January 1986 just because of the failure of an O-ring. Although in that case the accident was attributed to the use of rubber with improper glass-transition temperature (*T*_g_), it proves that failure of a polymeric material may cause great loss, or even disaster. Thus, accelerated aging in an air-circulating oven at elevated temperatures for months and monitoring changes of mechanical properties until failure for extrapolating to service temperature to obtain lifetime prediction is of great importance for aerospace, automotive, and petroleum industry applications [2,3]. Understanding the aging process when predicting the lifetime of elastomer components and foreseeing the timespan left until they have to be replaced is essential; generally, we can divide the aging process into physical and chemical aging. Understanding the main characteristics of the two aging typologies, the question discussed was how the aging of elastomers can be experimentally investigated. Direct or indirect method? On considering that oxidation is the dominant factor in a lot of polymer applications, a direct method like thermogravimetric analysis (TGA), differential scanning calorimetry (DSC), or dynamic mechanical analysis (DMA) are those most indicated [4,5,6,7,8].

The other investigated aspect was that of kinetic modeling of the degradation, which has lifetime prediction as its final goal. The different modeling approaches of the thermoanalytical data obtained with the main techniques, such as TGA, DSC, and differential thermal analysis (DTA), were discussed: from isoconversional (the reaction rate at constant extent of conversion is only a function of temperature) or model-free methods, which are generally split in two categories: differential and integral. The most common differential isoconversional method is that of Friedman [9,10], whilst examples of integral methods are the Ozawa–Flynn–Wall [11,12] and Vyazovkin [13] equations. On the other hand, we have the model-fitting methods (the derivation of kinetic parameters associated with a particular reaction model that is assumed to represent the conversion dependence of the reaction rate). Two of the most used are the Sestak–Berggren [14,15] and the Avrami–Erofeev [16,17] equations. In the middle ground is the Kissinger method [18], which yields a reliable estimate of activation energy only when conversion does not practically vary with heating rate. For this reason, it should not generally be called “isoconversional” [19,20].

Examining the literature of the last thirty–forty years, one comes across an unbridled use of the initial decomposition temperature (*T*_i_) and the activation energy of degradation (*E*_a_) to make considerations on the materials’ lifetime; at a more careful reading, a question arises spontaneously: which parameter must be considered to perform correct and reliable lifetime prediction of materials?

The most used parameter to determine the thermal stability of a material (and thus to perform a prediction on its lifetime) is certainly the *T*_i_ [21,22,23]. However, when we compare compounds with a *T*_i_ sufficiently close with each other, *E*_a_ must be considered [24,25,26], thus giving more importance to the determination of the kinetic parameters of degradation. Since experiments to obtain kinetic parameters of degradation are usually performed at temperatures near (or, in some cases, above) melting temperatures, the values obtained might not be suitable to represent the behavior of polymers in service, so the lifetime predictions based on these measurements could be questionable. Another question is added to the previous about the use of the most suitable parameter to make lifetime predictions: could one make a prediction relying exclusively on data obtained at high temperatures?

Furthermore, thermal degradation in polymeric materials occurs when the energy of vibration exceeds the primary bonding between atoms (thus, thermal stability is generally ascribed to the vibration energy of the chemical bonds), whilst the glass-transition phenomenon is related to the rotation and vibration of molecular chains [27]. According to these considerations and from the perspective of investigating a possible correlation between *T*_g_ and degradation, the temperature of isothermal experiments was gradually lowered to be close to the glass transition one.

Finally, but not less importantly, the aspects related to the disposal of plastics. The polymer-packaging industry is actually divided between the need to extend and implement principal packaging function containment, protection, and preservation, and the need to be easily disposed of or recycled after use. Thus, for these specific polymers used in food packaging, the study of their thermal properties is also crucial to improve recyclability or provide a viable alternative. In particular, lifetime predictions play a key role in facilitating both the design phase and the final disposition. But these predictions, up to today, have been based on the identification of the critical reaction that limits the life of a material; therefore, there was a strong need for accelerated lifetime characterization methodologies.

Before answering these questions, we have a certainty: the thermogravimetric (TG) technique is widely employed to determine kinetic parameters of polymer degradation. It has already been highlighted, as experiments carried out above the melting temperature of polymers, generally in dynamic condition, are usually performed to determine kinetic parameters associated with polymer degradation. Another problem is to give the correct physical meaning to the kinetics parameters used because the kinetics methods used to evaluate the TG data, obtained in the most different experimental conditions, are sometimes based on assumptions not always satisfied; thus, great care must be devoted before making actions towards polymer stabilization based on these results. 

With the aim to answer all of these questions, in this manuscript I have tried to correlate the experimental data obtained in the last 15 years by performing long- and short-term degradation measurements on several polymeric materials. The novelty of this procedure is represented by performing degradation experiments at temperatures lower than those usually employed, which were largely lower than the melting ones and, for a good part of the analyzed polymers, close to their *T*_g_. Degradation data obtained at these relatively low temperatures were compared to those obtained at higher temperatures in order to verify the reliability of lifetime predictions of polymers made by degradation experiments performed at higher temperatures (due to the time problem of the measure). Particular focus has been devoted to better investigating the role of the induction period (IP) in the degradation of complex systems, like polymers, and its effect on the prediction. 

## 2. Experimental

### 2.1. Materials

All used polymers are reported in Table 1, together with the code used for their identification. Polyetherketones (PEK, samples **1**–**3**) were synthesized according to the procedure previously reported [28] and were used in powder form. High-density polyethylene (PE, sample **4**), polystyrene (PS, sample **5**), polycarbonate (PC, sample **6**), and poly(methyl methacrylate) (PMMA, sample **7**), were standard for Gel Permeation Chromatography (GPC) products purchased from Fluka (Milan, Italy) and used without any further purification. PE, PS, and PMMA, which were in powder form, were used as received, while PC, which was in pellet form, was ground in a mortar before use. Polylactide (PLA, sample **8**) was a commercial food-packaging product and was used, in film form, as purchased without any special preliminary treatments. PE terephthalate (PET, sample **9**) and polypropylene (PP, sample **10**) were beverage- and food-packaging products and were used after washing and drying to remove any food or water debris. All polymers were dried under vacuum at room temperature and kept in a desiccator under vacuum until use. Their characteristics are reported in Table 2 together with the time of the long-term isothermal experiment.

### 2.2. Long-Term Isothermal Degradations

Long-term isothermal degradations were performed in static air atmosphere. Alumina crucibles containing exactly weighed quantities of the investigated polymers were put in an oven at different temperatures and times as a function of the polymer tested. Samples **1**–**3** at 270 °C for 37 months; samples **4**–**7** at 150 °C for 37 months; sample **8** at 150 °C for 9 months; and samples **9** and **10** at 150 °C for 25 months. 

Samples were weighed at specific time intervals using a Mettler AE 240 electronic balance (±1 × 10^−5^ g). Samples **1**–**3** were weighed every 30–40 days; samples **4**–**7** were weighed once a day (first two weeks of measurements), and then three times a week (for 1 month), twice a week (for another month), and successively once a week; sample **8** was weighed once a day (first 2 months of measurements), then three times a week (for 3 months), and successively once a week; samples **9**–**10** were weighed once a day (first 2 months of measurements), then 3 times a week (for 3 months), successively once a week (for 3 months), and finally once a month. To this aim, crucibles were extracted from the oven, cooled in a desiccator for 1 h, weighed, and then immediately put in the oven again. 

To avoid problems related with the frequent entry and exit from the oven, two sets of the same samples of about the same quantities were put inside the oven. One was used for measurements and the second one was left inside the oven throughout the experiment. At the end of the experiment, no remarkable difference in mass loss among the two sets of the same samples were recorded.

### 2.3. Short-Term Isothermal Degradations

A Mettler TA 3000 TG analyser, coupled with a Mettler TC 10A processor as control and evaluation unit (Mettler-Toledo, Greifensee, Switzerland), and a Shimadzu DTG-60 simultaneous DTA-TG apparatus (Shimadzu, Kyoto, Japan) were used for short-term degradations. The temperature calibration of TGA instruments was made according to the procedure suggested by the two companies and reported in previous works [23,29], based on the change of magnetic properties of 3 metal samples at their Curie points (isatherm, 142.5 °C; nickel, 357.0 °C; and trafoperm, 749 °C) for Mettler TGA and using indium (NIST SRM 2232), tin (NIST SRM 2220), and zinc (NIST SRM 2221a) as standard materials for temperature and a set of exactly weighed samples for mass for Shimadzu DTG. Isothermal experiments were performed in static air atmosphere in order to exactly reproduce the long-term degradation conditions.

Samples of about 5 × 10^−3^ g, held in alumina open crucibles, were heated (20 °C·min^−1^) from room temperature to the selected one and then maintained at this temperature for 900 min. The weight of the samples at the start of isothermal heating was considered the initial one. Degradations were repeated 3 times, and the *D* average values, where *D* = (*W*_0_ − *W*)/*W*_0_, and *W*_0_ and *W,* were the weights at the starting point and during scanning, at various times were in agreement with the experimental ones within ±3% in every case. 

### 2.4. Kinetics Method

The set of short-term experimental isothermal data was used to evaluate the *E*_a_ of our polymers through the MacCallum [30,31] method, based on the following linear equation:

ln*t* = a + b/*T*_iso_ (1)

where *t* was the time employed to reach a fixed degree of degradation *D*, a = ln[F(1 − *D*)] − lnA, b = *E*_a_/R, *T*_iso_ was the temperature of isothermal degradation, and F(1 − *D*) a function of degree of degradation. The apparent activation energy at each degree of degradation was obtained by the slope of the ln*t* vs. 1/*T*_iso_ straight line.

## 3. Results and Discussion

The study started with the isothermal degradation of 3 high thermally stable aromatic PEK (Table 1), samples **1**–**3**. A long-term experiment at a temperature, 270 °C, largely lower than the melting ones was carried out aiming to correlate data collected in these conditions with those obtained by short-term experiments performed at temperatures near the melting one. The long-term experiment was performed in an oven, whilst the short-term ones in a TG balance. An induction period of 1115, 418, and 150 days was observed for samples **1**, **2,** and **3** respectively, at 270 °C, whilst for the short-term experiments, no IP was recorded [28]. 

The different behavior observed for polymer degradation below melting and above it, in the molten state, had the consequence that, when we tried to insert the value obtained at low temperature into the kinetic equations obtained at a higher temperature with short-time experiments, a marked deviation of linearity was observed [28]. This obtained the first result, which means different degradation apparent activation energy in the two studied conditions suggested caution in the lifetime prediction performed by using data obtained at high temperatures. 

Among the questions that arose spontaneously after these first results were those related to the typologies of used materials, very thermally stable polymers, and to the temperature chosen for the long-term experiment, which was, however, only relatively low. Consequently, the next step was to repeat the comparison between long- and short-term experiments using, this time, four well known polymers, PE, PS, PC, and PMMA, and a much lower isothermal temperature, 150 °C. It is worth to note that, for three of the used polymers, the chosen temperature is very close to their *T*_g_. 

**Figure 2:**
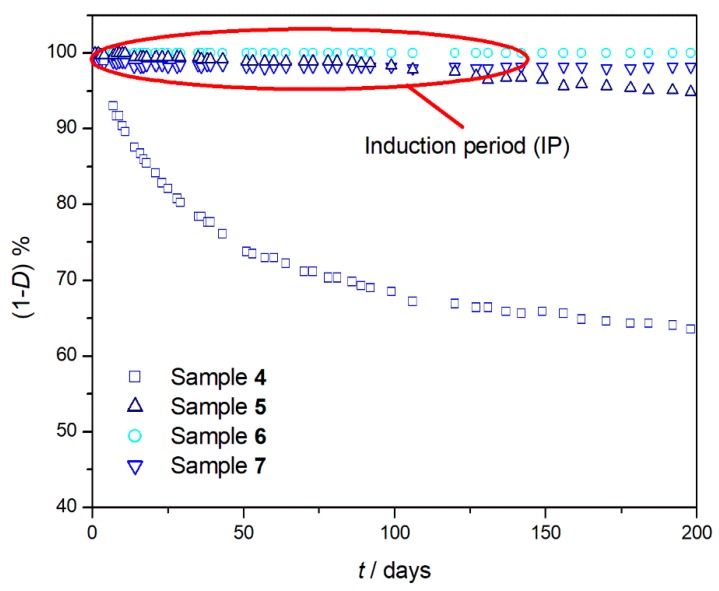
Percentage of undegraded sample (1 − *D*)%, at 150 °C as a function of heating time (*t*) for PE (sample **4** □), PS (sample **5** △), PC (sample **6** ○), and PMMA (sample **7** σ) after 200 days of degradation.

Whilst for PS (sample **5**), PC (sample **6**), and PMMA (sample **7**), whose *T*_g_ is very close to the isothermal one (Table 2), a long IP was observed (Figure 2), for PE (sample **4**), whose *T*_g_ is more lower [32], no IP was observed.

**Figure 3:**
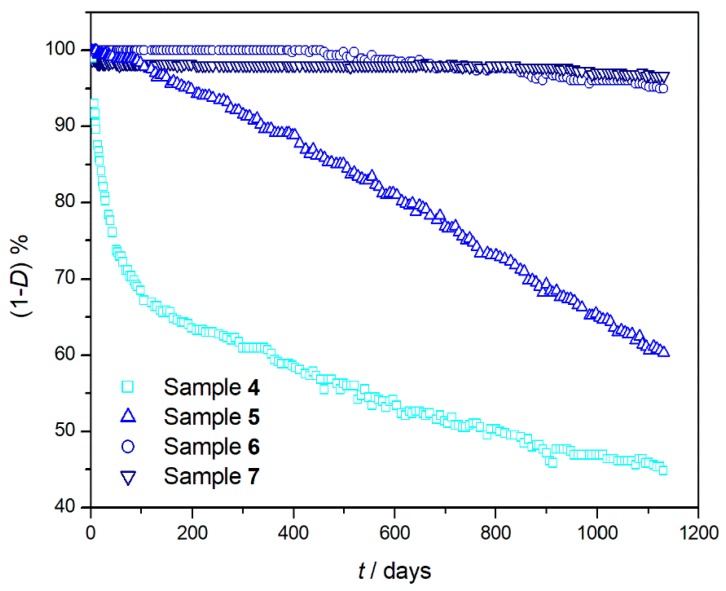
Percentage of undegraded sample (1 − *D*)% at 150 °C as a function of heating time (*t*) for PE (sample **4** □), PS (sample **5** △), PC (sample **6** ○), and PMMA (sample **7** σ) after 1130 days of degradation.

On considering that the IP of PC and PMMA lasted for almost the entire time of the experiment, the comparison between long- and short-term experiments was possible only for the two polymers that have significantly lost weight during the long-term experiment, i.e., PE and PS (Figure 3). 

**Figure 4:**
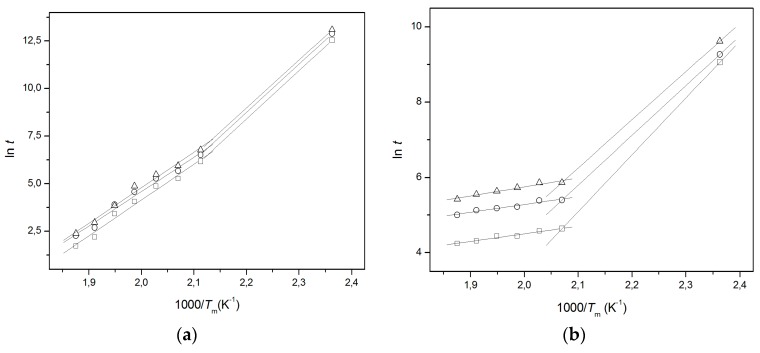
MacCallum straight lines at various *D* values: 5% (□), 7.5% (○), 10% (△) for (**a**) PE and (**b**) PS.

No matching among the kinetics data obtained by using the MacCallum equation was observed (Figure 4) for both polymers, but when the IP was not present, PE (Figure 4a), the mismatching was quite different in respect to the PS (Figure 4b) that showed an IP of about 150 days. The same behavior was observed by treating the kinetic data, obtained in the short-term experiments, with other kinetics methods as well [33,34]. 

After all these years of long-term experiments, the first conclusion was that lifetime predictions of polymers in service made by degradation experiments at higher temperatures seems not reliable. The possibility to find useful structure–IP correlations began to make its way. 

After studying very stable synthetic polymers (samples **1**–**3**) and model polymers (samples **4**–**7**), to go ahead, with the aim to strength this structure–IP correlation, the choice fell on two different commercial PLA (samples **8a** and **8b** in this manuscript) used for food packaging [35]. PLA was selected for two different reasons:

- Despite the advertizing, its recycling is not economically viable and, since it is one of the most used polymeric materials in food packaging because of its ability to be degraded, it represents one of the major domestic waste materials; thus, it could be interesting and useful to predict, at least roughly, its end life in the environment.
- On considering the original purpose of the work to verify the validity of lifetime predictions by extrapolating the kinetics data obtained at high temperatures, PLA was chosen because of its low glass transition and melting temperature. In respect to the polymers examined in the past, it suggested the possibility of a sizable degradation in a reasonable time during long-term measurements.

**Figure 5:**
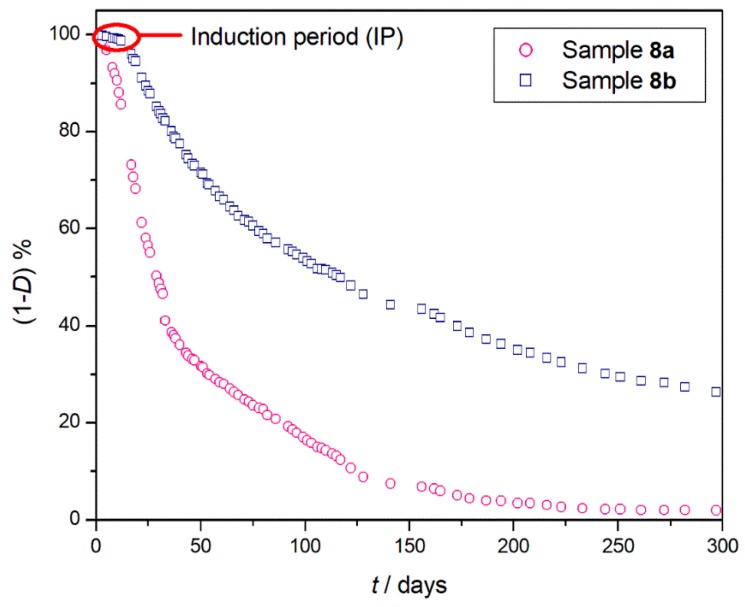
Percentage of undegraded sample (1 − *D*)% at 150 °C as a function of heating time (*t*) for PLA (sample **8a** ○) and (sample **8b** □) after 300 days of degradation.

Sample **8a** started to degrade immediately, differently than compound **8b**, which showed an initial IP of about ten days. As expected, the degradation in this case was faster and the two PLA samples, which reached almost complete degradation after just 300 days (Figure 5), a third of the time needed by the polymers previously examined (samples **1**–**7**), to achieve a much lower degradation percentage. The degradation data obtained during the long-term experiment were also in this case compared with those obtained from the short-term one, and a slightly mismatching was observed for both investigated samples [35]. 

In order to quantify the uncertainty in the lifetime prediction of the studied polymers, a comparison among the measured trend of the real degradation and that extrapolated from the MacCallum equations has been performed, showing about twelve days of difference for sample **8a** and about sixteen days for sample **8b** [35]. It is worth to note that also in this case, where no IP was present, the real and the expected values were closer than the polymer for which an IP (although the slightest) is present.

**Figure 6:**
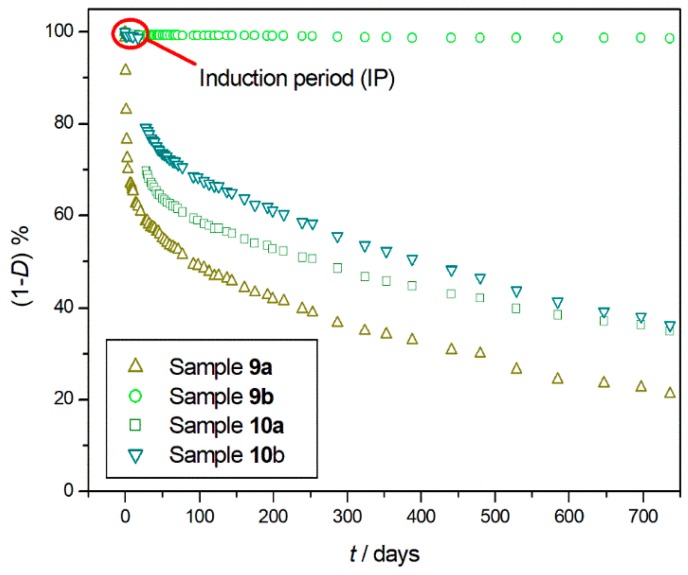
Percentage of undegraded sample (1 − *D*)% at 150 °C as a function of heating time (*t*) for PET (sample **9a** △ and **9b** ○) and PP (sample **10a** □ and **10b** σ) after 750 days of degradation.

On continuing the research in this direction, two different types of commercial PET (samples **9a** and **9b**) and PP (samples **10a** and **10b**) used for beverage and food packaging were degraded for more than two years in isothermal oxidative condition at 150 °C [36]. Long-term degradation showed that sample **9a** started to degrade immediately without IP, differently than samples **10a** and **10b**, which both showed a short IP of about twenty days. Sample **9b** did not show appreciable mass loss and, thus, IP for the whole time of the experiment (Figure 6). The overall mass losses, in about 750 days, were 1.5, 79, 64, and 65% for polymers **9a**, **9b**, **10a,** and **10b,** respectively. According to the practice followed for the other polymers previously investigated, the data acquired from the long-term experiments were compared with thermo-oxidative degradations, carried out on the same materials at higher temperatures. The comparison was obviously performed for the polymers (samples **9a**, **10a,** and **10b**) that showed appreciable mass loss during the long-term experiment.

**Figure 7:**
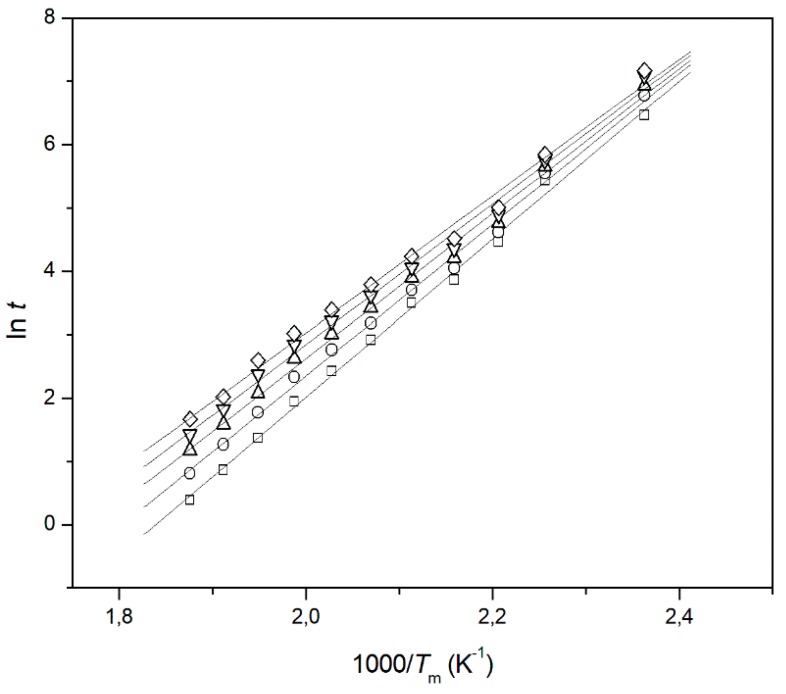
MacCallum straight lines at various *D* values: 5% (□), 7.5% (○), 10% (△), 12.5% (σ), and 15% (◇) for Sample **9a**.

In the case of sample **9a** for the first time, differently than what we found for all the other experiments, the comparison of the long-term experimental data with those obtained in short-term modality resulted in a perfect match (Figure 7). For the other samples, expectations have also been confirmed.

For sample **9b**, which presented a very long IP at 150 °C, no matching was possible. Instead, for samples **10a** and **10b**, whose IPs were very short, a difference of the order of nine days was recorded in the comparison with real data, highlighting the hypothesis that the IP greatly influences the lifetime predictions achieved by extrapolating data obtained in the molten state.

**Figure 8:**
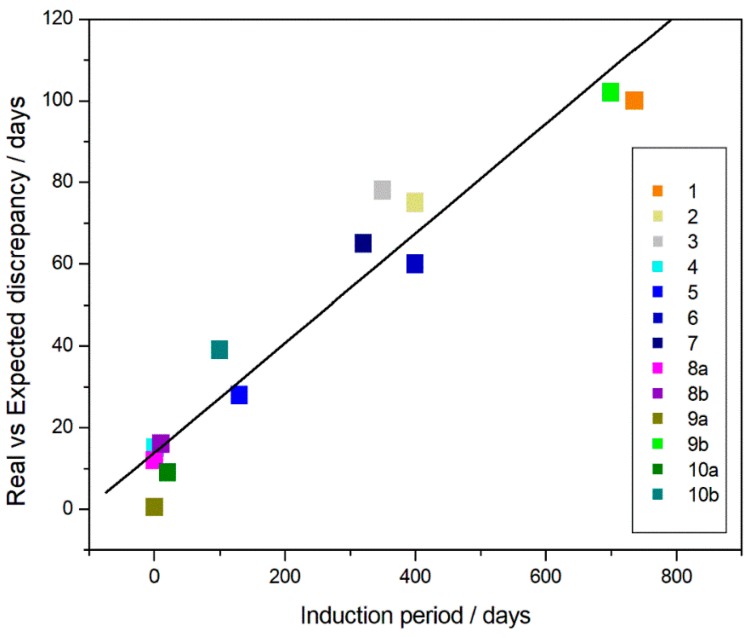
Relationship between the discrepancy (among the real and expected data) and the IPs observed in the long-term degradation experiments.

Is the discrepancy between real and expected data (RE) dependent on the length of IP? If we look at Figure 8, where this discrepancy is reported as a function of the IPs obtained from the various experiments performed with different polymers, the existence of a relationship appears clear and probably will help those people who are approaching to realize lifetime predictions by using the IP length to correct, according to the below equation:

RE = 13.9 (±4) + 0.134 (±0.01) IP(2)

the extrapolated data from measurements performed in the molten state.

## 4. Conclusions

The time to answer the questions made at the beginning of this long work and in the introduction of this article has come: which parameter must be considered to perform correct and reliable lifetime prediction of materials? Could one make a prediction relying exclusively on data obtained at high temperatures?

If, on the one hand, experimental experience and the literature confirm the importance of the calculation of the *E*_a_ of degradation to perform a lifetime prediction, the work carried out in these years and, above all, the correlations here put in place between the various data obtained, with different polymers and at different temperatures, from long-term experiments and the relative IPs, prove that we can not disregard this latter parameter in lifetime predictions. In particular, a relationship to help people in correcting the data obtained by TGA experiments performed at high temperatures is proposed.

## Figures and Tables

**Table 1:** Molecular formula of the tested polymeric materials.

Sample Code	Molecular Formula	Abbreviation
**1**	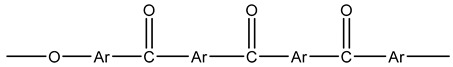	PEEK1
**2**	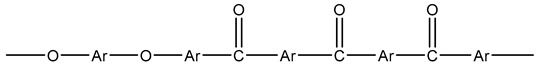	PEEK2
**3**	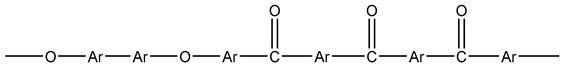	PEEK3
**4**	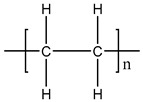	PE
**5**	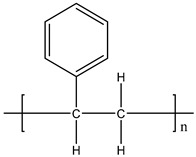	PS
**6**	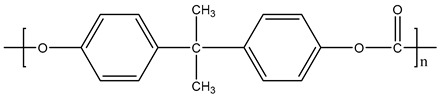	PC
**7**	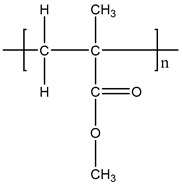	PMMA
**8**	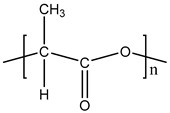	PLA
**9**	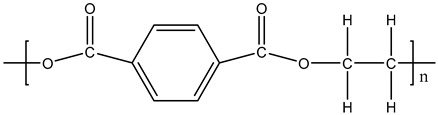	PET
**10**	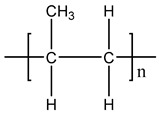	PP

where: Ar=1,4-substituted phenylene.

**Table 2:** Sample code, abbreviation, molecular weight (*M*_w_), glass-transition temperature (*T*_g_), onset melting temperature (*T*_onset_), and time of long-term experiment of the tested polymeric materials.

Sample Code	Polymer	*M*_w_	*T*_g_ °C	*T*_onset_ °C	Ref.	Experimental Time/Months
1	Polyetherketones 1	n.a.	n.d.	417.0	[27]	37
2	Polyetherketones 1	n.a.	n.d.	379.0	[27]	37
3	Polyetherketones 1	n.a.	n.d.	434.0	[27]	37
4	Polyethylene (PE)	60,000	n.a.	129.2	[31]	37
5	Polystyrene (PS)	65,000	99.5	n.d.	[31]	37
6	Polycarbonate (PC)	50,000	145.9	n.d.	[31]	37
7	Poly(methyl methacrylate (PMMA)	65,000	118.1	n.d.	[31]	37
8a	Polylactide (PLA) 1	n.a.	60.0	140.4	[33]	9
8b	Polylactide 2	n.a.	63.5	139.9	[33]	9
9a	Polyethylene terephthalate (PET) 1	n.a.	76.9	229.2	[34]	25
9b	Polyethylene terephthalate 2	n.a.	33.0	140.8	[34]	25
10a	Polypropylene (PP) 1	n.a.	47.8	151.0	[34]	25
10b	Polypropylene 2	n.a.	58.9	131.6	[34]	25
